# Inter-brain coupling during naturalistic collaborative Tangram solving in child dyads: an exploratory fNIRS hyperscanning study

**DOI:** 10.1007/s00429-026-03187-5

**Published:** 2026-08-29

**Authors:** Raimundo da Silva Soares Junior, João Ricardo Sato

**Affiliations:** 1 Center for Mathematics, Computation and Cognition (CMCC), Federal University of ABC (UFABC), São Bernardo do Campo, Brazil; 2 D’Or Institute for Research and Education, Rio de Janeiro, Brazil

**Keywords:** fNIRS hyperscanning, Child development, Peer collaboration, Embodied cognition, Ecological validity

## Abstract

Collaborative problem solving plays a central role in children’s cognitive and social development, yet how neural activity relates to real-time peer coordination remains poorly understood. Using functional near-infrared spectroscopy (fNIRS) hyperscanning, we simultaneously recorded brain activity from 15 dyads of school-aged children during a naturalistic Tangram puzzle task, including rest, solo problem-solving, observation, and collaboration. We examined task-related neural activation and inter-brain connectivity (IBC) to assess how interpersonal neural dynamics relate to task context and behavior. No prespecified region-of-interest activation effect survived correction for multiple comparisons. At the exploratory channel level, a posterior-parietal channel showed a positive HbO response during collaboration relative to baseline. The global IBC did not differ significantly across experimental conditions, indicating that inter-brain synchrony was not determined solely by collaboration or social presence. Nevertheless, an exploratory association between global HbO coupling during Duo and total manual activity showed a relevant magnitude (Spearman rho = 0.581), a bootstrap 95% confidence interval excluding zero [0.038, 0.891], positive coefficients in every leave-one-dyad-out analysis, and a similar magnitude after excluding the first 8 s of each block (rho = .538), although it did not meet the FDR threshold across the broader sensitivity family, q = 0.131. Building on the emerging literature on dual-child portable fNIRS hyperscanning, the study reinforces the viability of this approach in an ecologically valid, hands-on task and suggests that block-wise inter-brain coupling may capture meaningful variation in dyad-level behavioral engagement.

**Supplementary Information:**

The online version contains supplementary material available at https://doi.org/10.1007/s00429-026-03187-5.

## Introduction

Human cognition develops within deeply social environments. From early childhood, learning, problem-solving, and reasoning often occur through collaboration with others, in which shared attention, coordinated actions, and communication influence cognitive development (Rogoff 2003; Tomasello 2019). Collaborative problem solving is a key aspect of childhood social interaction and has been connected to improvements in executive function and academic progress (Kyndt et al. 2013; Warneken 2018; Doebel 2020). In fact, meta-analytic evidence indicates that collaborative goal structures and face-to-face collaborative learning are associated with higher academic achievement and more positive peer relationships than competitive or individualistic structures (Roseth et al. 2008; Kyndt et al. 2013). Despite the behavioral evidence, the neural processes underlying children's coordination of thoughts and actions during collaboration remain poorly understood. Understanding how children’s brains synchronize during real-world interactions is thus a challenge for developmental social neuroscience.

At the core of these real-world interactions is joint action (i.e., collaborating toward a shared goal). It requires partners to represent a shared goal, monitor one another’s behavior, predict upcoming actions, and adjust their own responses in real time (Sebanz et al. 2006; Vesper et al. 2010). Neuroimaging evidence suggests that the dorsolateral prefrontal cortex supports the maintenance of task goals, planning, working-memory operations, and the flexible control of behavior (Miller and Cohen 2001). In parallel, posterior parietal regions integrate visual, spatial, and somatosensory information with action planning and are particularly relevant for reaching, grasping, and object manipulation (Culham and Valyear 2006). Collaborative problem solving may also recruit processes involved in representing and updating information about a partner. The temporoparietal junction (TPJ) is a component of the mentalizing network and is engaged when individuals distinguish their own representations from those of others and reason about beliefs, intentions, and perspectives (Saxe and Kanwisher 2003; Schurz et al. 2014). Functional specialization within this network, including the right TPJ (rTPJ), is already evident in school-aged children and continues to develop throughout childhood (Saxe et al. 2009; Gweon et al. 2012).

Hyperscanning, a technique that records neural activity from two or more individuals simultaneously, provides a unique view of the neural processes underlying social interaction (Montague et al. 2002; Babiloni and Astolfi 2014). Instead of inferring social processes from isolated brains, hyperscanning allows measurement of inter-brain synchrony, showing how neural dynamics become aligned during communication, joint attention, and collaborative behavior. A solid body of adult research shows increases in inter-brain coupling during cooperation (Shaw et al. 2023), verbal communication (Jiang et al. 2012), coordinated movement (Konvalinka and Roepstorff 2012), and shared attention (Dumas et al. 2010). Such synchrony has been interpreted as reflecting interpersonal coordination processes, which are key parts of social cognition (Hasson et al. 2012; Redcay and Schilbach 2019).

In adult hyperscanning literature, a critical distinction is often drawn between co-action (i.e., performing tasks side by side) and joint action. Evidence suggests that physical proximity or simultaneous action alone is insufficient to elicit robust inter-brain synchrony; rather, shared intentionality and reciprocal coordination drive neural alignment (Cui et al. 2012; Cheng et al. 2015). However, it remains unclear whether this dissociation applies to children. Since childhood development is heavily characterized by parallel play and observational learning, determining whether children's neural synchrony emerges from social presence or requires active behavioral engagement is a key open question.

To date, developmental hyperscanning research remains comparatively limited and has predominantly examined interactions between children and adults, including parent–child communication, emotional co-regulation, joint attention, and teaching contexts (Reindl et al. 2018; Bi et al. 2023; Hakim et al. 2023; Alonso et al. 2024). Peer interactions are theoretically distinct because both partners are undergoing cognitive and social development and may contribute more symmetrically to the interaction. Emerging child-child hyperscanning studies suggest that interpersonal neural coupling may vary as a function of peer relationship, interaction context, and the behavioral demands of collaboration or competition (Bonnaire et al. 2024; Zhou et al. 2025; Nelson et al. 2025). Although functional near-infrared spectroscopy (fNIRS) offers an ideal modality for ecologically valid settings due to its tolerance for movement (Piazza et al. 2020; Oku et al. 2022, 2023), there remains a critical need to explore embodied collaborative problem-solving. Specifically, it is crucial to understand how inter-brain dynamics, and potentially their underlying network topology (Czeszumski et al. 2021) operate when child peers engage in naturalistic, hands-on problem-solving that requires continuous object manipulation and mutual behavioral adjustment.

The primary objective of the present study was to investigate the inter-brain dynamics of child dyads during an ecologically valid, hands-on collaborative task, aiming to contrast active joint engagement with individual action, observation, and rest. To bridge the gap between strictly controlled experiments and the dynamic nature of peer interaction, we employed fNIRS hyperscanning during a naturalistic Tangram puzzle task. Grounded in embodied cognition theories, which posit that cognitive processes are rooted in physical interaction (Glenberg 2008), our experimental design contrasted rest, individual action, observation of a partner, and collaborative manipulation. By tracking simultaneous activity in dorsolateral prefrontal, posterior parietal, and temporoparietal regions, selected for their respective roles in executive control, visuomotor integration, action monitoring, and social cognition (Saxe and Kanwisher 2003; Barreto et al. 2021; da Silva Soares et al. 2024), we sought to determine whether joint cognitive performance inherently covaries with the physical dynamics of manual action.

Based on this framework, we tested three specific hypotheses: First, we expected collaborative problem solving to elicit greater activation in posterior parietal and dorsolateral prefrontal regions than rest, reflecting the increased demands of visuomotor integration, goal maintenance, and behavioral adjustment. We also examined the rTPJ as a social-cognitive region of interest (ROI), expecting that conditions involving attention to a partner’s actions would engage this region more strongly than rest. Second, we expected inter-brain coupling to be greater during collaboration than during rest, individual action, or observation, positing that true collaboration requires reciprocal behavioral adjustment beyond simple co-presence or exposure to a shared task. Third, we hypothesized that dyads with greater overall task-related manual engagement would show stronger global HbO inter-brain coupling during collaboration. Given the limited child-child literature and the sample of 15 dyads, the neural analyses were explicitly treated as exploratory and were evaluated using dyad-aware inference, effect sizes, confidence intervals, multiple-comparison control, pseudodyads, and sensitivity analyses.

## Methods

### Participants

The study received approval from the local ethics committee, and written informed consent was obtained from parents/guardians and children's assent. The study recruited 30 children who formed 15 dyads (18 boys and 12 girls; mean age = 9.47 years, SD = 1.61, median = 9, range = 6–12 years). Mean ages were 9.17 years (SD = 1.76) for boys and 9.92 years (SD = 1.31) for girls. Eight dyads were mixed-sex, five were male-male, and two were female-female. The mean absolute within-dyad age difference was 1.33 years (SD = 1.29, median = 1, range = 0–4). Children with diagnosed neurological or psychiatric conditions were excluded. Participants were recruited from the local school community, and all partners were classmates who knew one another before testing. Dyads were formed by convenience, based on participant availability.

### Task: Tangram puzzle

The Tangram task models collaborative problem solving, frequently used in early education to promote spatial reasoning and shared strategy formation (Ayaz et al. 2012). Each Tangram kit comprised seven colored geometric pieces forming a 14.2 cm × 14.2 cm square. Twenty-four target configurations were presented sequentially on an iPad, and the children reproduced each configuration by manipulating the physical pieces on the table. This setup required children to translate the two-dimensional visual template into a physical arrangement and therefore engaged visuospatial analysis, object manipulation, and action planning (Ayaz et al. 2012; da Silva Soares et al. 2024). If a pair failed to solve a puzzle within four minutes, the experimenter advanced to the next challenge. This approach was designed to ensure engagement while maintaining a suitable level of challenge.

### Experimental Procedure

**Fig. 1:**
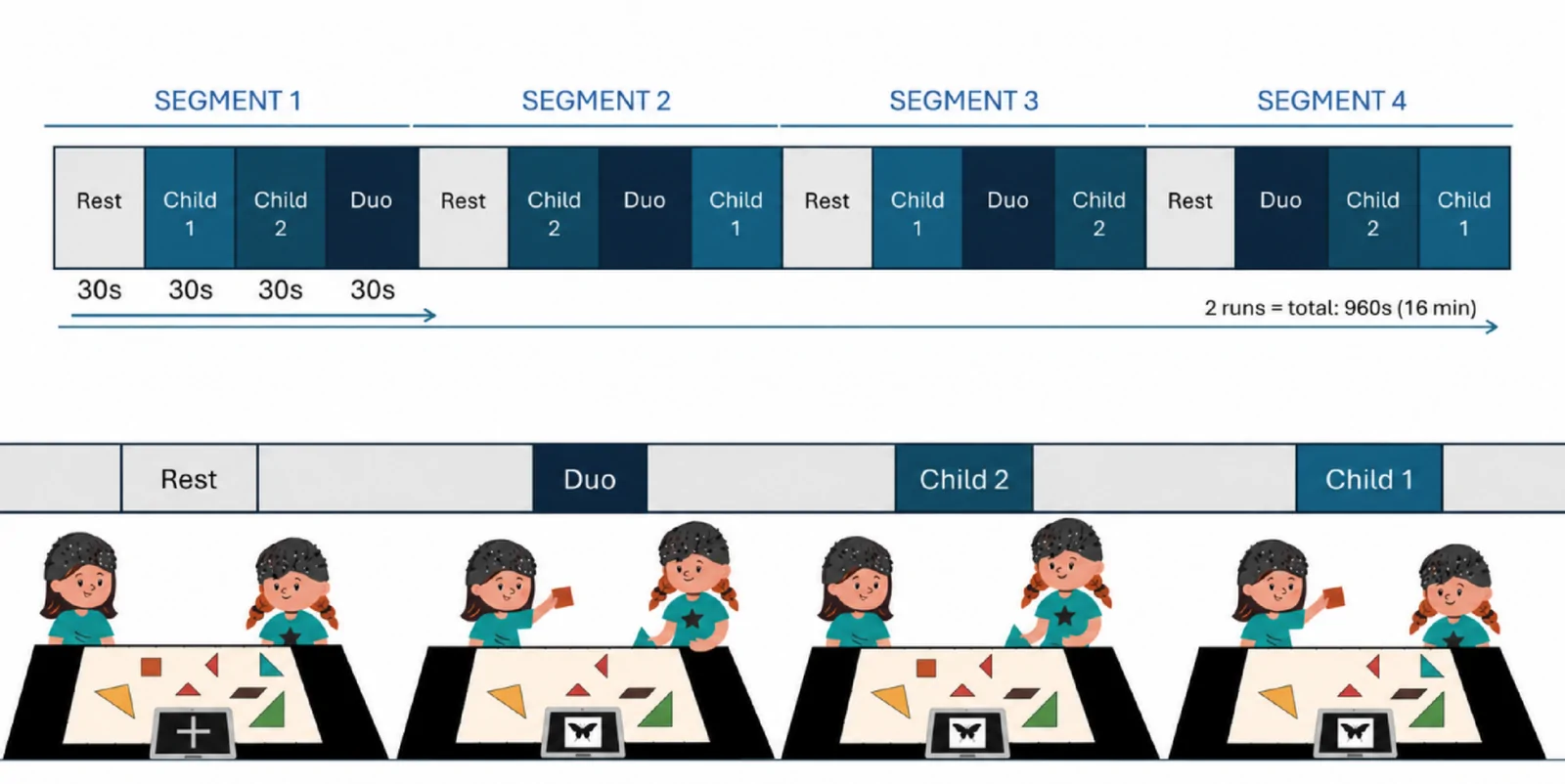
Experimental design of the child–child Tangram hyperscanning task. Illustration of the experimental setup showing two children seated side-by-side wearing fNIRS caps during the Tangram task. Each run comprised four segments, with each segment containing four 30-s blocks corresponding to Rest, Child 1, Child 2, and Duo blocks in a predetermined order. During individual blocks, only the designated child manipulated the pieces; during Duo, both children collaborated. Two runs yielded a total acquisition time of 960 s

Each pair participated in one session lasting approximately 25 min. Children sat side by side with clear visibility of the tablet and workspace and were randomly assigned to the right or left seat, corresponding to the labels Child 1 and Child 2. A camera directed toward the Tangram pieces and tablet recorded manual behavior. Participants completed one collaborative practice model before fNIRS acquisition to ensure that both children understood the puzzle and condition-specific instructions. Prerecorded audio cues indicated the current condition. During Child 1 blocks, only Child 1 was permitted to manipulate the pieces while Child 2 observed; during Child 2 blocks, the roles were reversed. During Duo blocks, both children could manipulate the shared pieces to reproduce the model. During Rest, both children fixated on a cross displayed on the iPad. Children were instructed not to speak or use intentional gestures during any recorded condition. The experimental setup and command structure are illustrated in Fig. 1.

The protocol comprised two runs, each containing the same four predetermined four-block segments. Segment 1 followed Rest-Child 1-Child 2-Duo; Segment 2 followed Rest-Child 2-Duo-Child 1; Segment 3 followed Rest-Child 1-Duo-Child 2; and Segment 4 followed Rest-Duo-Child 2-Child 1. Each block lasted 30 s, and the four-segment sequence was repeated in the second run, yielding eight blocks per condition and a total acquisition time of 960 s. Condition position therefore varied across segments, but block order was not independently randomized for each dyad. When a puzzle was solved, or the four-minute limit was reached, the experimenter presented the next model. The experimental design is displayed visually in Fig. 1.

### fNIRS data acquisition

**Fig. 2:**
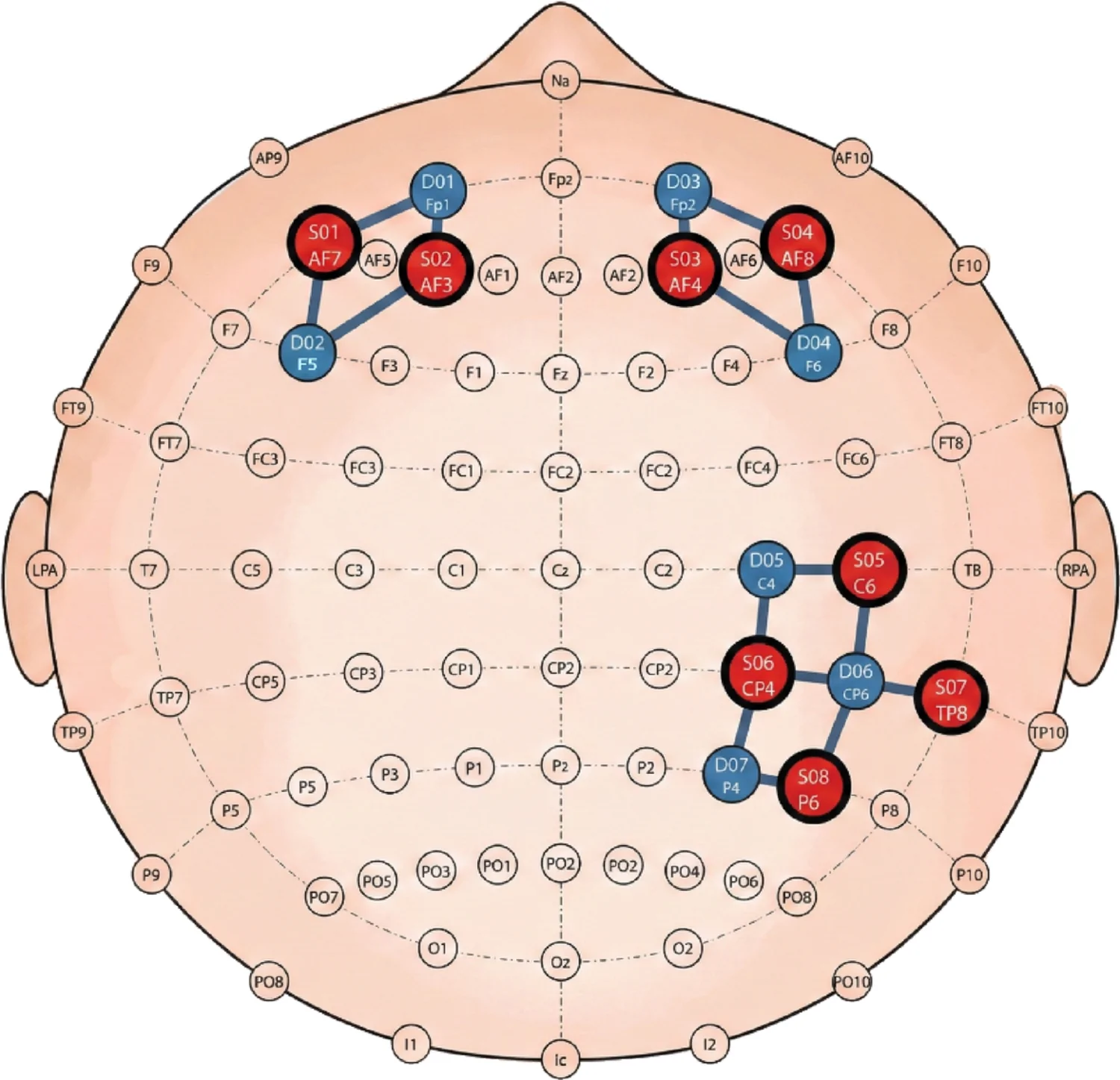
Optode placement covered the prefrontal cortex and the right temporoparietal regions based on the 10–20 EEG system using a cap with holders. Blue circles represent detectors, red circles represent sources, and black circles indicate short-separation detectors. Source–detector pairs forming long-separation channels are connected by blue lines

fNIRS signals were acquired using a portable NIRSport2 system (NIRx, Germany), with an acquisition sampling rate of 10.17 Hz, 16 emitters and 16 detectors (continuous wave), split between the two participants. Calibration and acquisition were performed using Aurora software (NIRx Medical Technologies, Germany). Optode placement (Fig. 2) covered the frontal and parietal regions, based on the international 10–20 EEG system, using a cap with holders. Short-distance channels (one per source bundle) were used to regress out potential systemic artifacts. Regions of interest (ROIs) were defined as the right posterior parietal regions (channel 14 and 17) (Koessler et al. 2009), associated with reaching and grasping coordination, dorsolateral prefrontal cortex (dlPFC, channels 2, 5, 8, 11) related to spatial cognition (da Silva Soares et al. 2024) and the temporoparietal junction (TPJ, channels 15, 18, 21, 23) often implicated in social cognition in prior imaging literature (Barreto et al. 2021).

### fNIRS data analysis

The data were preprocessed in Satori v1.8 software (Brain Innovation, the Netherlands). The modified Beer-Lambert law was applied to convert the optical signals of each wavelength (760 and 850 nm) into concentration changes of oxy-Hb and deoxy-Hb. Pre-processing routines included motion correction using Temporal Derivative Distribution Repair (TDDR) and high-frequency restoration. This process also involved spike removal and short-channel regression, in which a general linear model was fitted, using the highest-correlated short channel as a regressor for each channel signal. Additionally, temporal filtering was applied using a high-pass Butterworth filter at 0.01 Hz and a low-pass filter at 0.50 Hz to eliminate slow drift and high-frequency noise, such as systemic artifacts (Cui et al. 2010; Tachtsidis and Scholkmann 2016; Yücel et al. 2021).

First-level general linear models were estimated separately for HbO and HbR using a canonical hemodynamic response function (Huppert 2016). The two solo conditions were relabeled according to each child's role: Active denoted blocks in which that child manipulated the pieces, whereas Observation denoted blocks in which the partner manipulated the pieces. Contrasts were Active > baseline, Observation > baseline, Duo > baseline, Duo > Active, Duo > Observation, and Active > Observation. To avoid treating members of the same dyad as independent observations, participant beta estimates were averaged within dyad before confirmatory group inference (N = 15 dyads).

Three regions of interest (ROIs) were specified from the montage and theoretical hypotheses: dorsolateral prefrontal cortex (channels 2, 5, 8, and 11), right posterior parietal cortex (channels 14 and 17), and right temporoparietal junction (channels 15, 18, 21, and 23). Participant beta estimates were averaged within dyad before confirmatory inference, yielding N = 15 independent dyad-level observations. ROI means were tested against zero with Holm correction across the three ROIs within each contrast. Channel-wise tests across all 24 GLM channels were FDR-corrected and treated as exploratory. Exact sign-flip tests, bootstrap confidence intervals, participant-level sensitivity analyses, and leave-one-dyad-out results were also calculated. The same framework was applied to HbR, and HbO-HbR convergence was evaluated from effect direction and dyad-level associations. The exploratory channel-17 results and sensitivity checks are reported in Supplementary Table S3.

### Behavioral coding

Video recordings were reviewed offline by a single investigator using an event-logging approach. Task-related manual activity for each participant (designated by seating position, e.g., right or left partner) was manually annotated. Each continuous movement was recorded with precise onset and offset timestamps to define discrete manual-action episodes. To align the behavioral data with the neuroimaging time series, a fixed 30-s temporal shift was applied, anchoring the video to the first active block. Episode frequency and cumulative duration were then calculated for each condition; episodes crossing a 30-s block boundary were proportionally divided for duration analyses. Because a single Tangram puzzle could span multiple conditions, puzzle completions were retained strictly as session-level measures. Finally, an absolute contribution-asymmetry index, calculated as |right-left|/(right+left), was used to quantify the behavioral imbalance between partners. Dyad-level behavioral values are provided in Supplementary Table S1.

### Preprocessing of fNIRS time series

For each dyad, raw oxyhemoglobin (HbO) signals were imported separately for each participant and processed according to the analysis pipeline described in the supplementary material. Sixteen long-distance channels were selected for functional connectivity analysis. To ensure comparability across individuals and dyads, all HbO time series were standardized using within-participant z-scoring. Standardized signals were then organized into two three-dimensional matrices, with dimensions of 15 dyads × 9,766 time points × 16 channels. This structure enabled the subsequent concatenation of channels across participants, which was required for the connectivity analysis.

### Inter-brain connectivity computation

For each participant, the 16 retained long-separation channels were standardized across the complete recording. A 16 × 16 cross-brain Pearson correlation matrix was calculated separately for every 30-s block. Each coefficient was transformed using Fisher's r-to-z transformation before averaging across the eight repetitions of each condition, and negative correlations were retained. Global inter-brain coupling (IBC) was defined as the mean of all 256 transformed cross-brain coefficients, replacing the raw sum used in the initial analysis. Additional summaries represented the 16 homologous channel pairs and within- and between-region connections involving the dorsolateral prefrontal, posterior-parietal, and right temporoparietal ROIs. The primary estimand was zero-lag cross-brain covariance within the experimentally defined 30-s conditions. Pearson correlation was selected because its block-wise computation preserves direct alignment with the audio commands and triggers. Complete condition-level, regional, and channel-pair results are provided in Supplementary Table S4 and Supplementary Data S1.

All Pearson analyses were repeated using the full 30-s blocks and after excluding the first 8 s of every block. The 8-s exclusion was selected because behavioral coding showed that 95% of manual activity assigned to Rest occurred within the first 7.8 s, consistent with short-lived transition behavior. Temporally matched pseudodyads were generated by pairing Child 1 from each real dyad with Child 2 from a different dyad using 5,000 random derangements. This procedure preserved the full experimental sequence and condition timing while disrupting the real partnership. Real-pseudodyad results are reported in Supplementary Table S5; carry-over sensitivity results are reported in Supplementary Table S6 and Supplementary Figure S1.

### Statistical analysis

Behavioral condition effects were evaluated with Friedman tests across Child 1, Child 2, and Duo, followed by Holm-corrected Wilcoxon signed-rank comparisons. Connectivity condition effects were assessed using Friedman tests and Kendall's W. Omnibus p-values were controlled for using the Benjamini–Hochberg false discovery rate across the prespecified connectivity summaries within each chromophore and trimming strategy. Pairwise Wilcoxon tests were Holm-corrected within each summary, and channel-pair contrasts were FDR-corrected across 256 comparisons. Real-pseudodyad differences were evaluated against permutation null distributions. Brain-behavior associations were assessed using Spearman correlations with 10,000-permutation p-values, percentile-bootstrap 95% confidence intervals based on 5,000 resamples, leave-one-dyad-out sensitivity analyses, and FDR correction within each condition. Sensitivity analyses estimated the minimum detectable standardized mean effect and correlation at 80% power (Supplementary Table S9).

## Results

### Dual-child fNIRS hyperscanning data completeness

The sample comprised 30 children (18 boys and 12 girls; mean age = 9.47 years, SD = 1.61) in 15 dyads. Eight dyads were mixed-sex, five were male-male, and two were female-female; the mean absolute within-dyad age difference was 1.33 years (SD = 1.29). All dyads completed the full 16-min fNIRS protocol, and no datasets were excluded due to technical failure or unusable signal.

### Collaboration produced a behaviorally distinct task state

Dyads solved an average of 4.13 puzzles (SD = 2.20, median = 3, range = 2–10) and produced 229.67 manual-action episodes on average (SD = 39.15). Right- and left-side action counts were similar, t(14) = 0.85, p = 0.410, dz = 0.22, and the median absolute contribution asymmetry was 0.13 (IQR = 0.05–0.18). Descriptive values are reported in Table 1 and Supplementary Table S1.

**Table 1:** Condition-specific manual activity across dyads

Condition	Action episodes, M (SD)	Duration, s, M (SD)
Child 1	61.13 (11.79)	130.32 (22.17)
Child 2	64.47 (10.82)	133.73 (14.38)
Duo	94.60 (21.70)	158.27 (27.54)

Manual-action frequency differed across active conditions, Friedman chi-square(2) = 22.80, p < 0.001, Kendall's W = 0.76. Duo blocks contained more action episodes than Child 1 (Holm-adjusted p = 0.002, dz = 2.12) and Child 2 blocks (adjusted p = 0.002, dz = 2.02), whereas the two individual-action conditions did not differ (adjusted p = 0.244). Action duration also differed across conditions, chi-square(2) = 14.53, p < 0.001, W = 0.48. Thus, the collaborative condition was behaviorally distinct rather than a nominal relabeling of the same task state (Table 1).

### Task-related neural activation

Confirmatory dyad-level ROI analyses did not identify an HbO effect that survived Holm correction for any contrast. For Duo > baseline, the posterior-parietal ROI showed a positive but nonsignificant HbO estimate, M = 18.24, SD = 61.48, t(14) = 1.15, p = 0.270, Holm p = 0.654, dz = 0.30, 95% CI [− 15.81, 52.29]. The corresponding HbR ROI estimate was directionally negative but nonsignificant, M = − 1.15, SD = 16.32, t(14) = − 0.27, p = 0.789. Complete ROI results are presented in Supplementary Table S2.

At the exploratory channel level, channel 17 showed a positive Duo > baseline HbO effect, M = 21.14, SD = 37.99, t(14) = 2.16, uncorrected p = 0.049, FDR q = 0.594, dz = 0.56, 95% CI [0.11, 42.18]. HbR at the same channel was negative on average, M = − 9.44, t(14) = − 1.70, p = 0.110, and HbO and HbR estimates were inversely associated across dyads, rho = − 0.76, p = 0.001. However, the HbO result did not survive channel-wise correction, and leave-one-dyad-out p values ranged from 0.035 to 0.098. The full estimates are presented descriptively in Supplementary Table S3.

### Inter-brain connectivity across task conditions

**Fig. 3:**
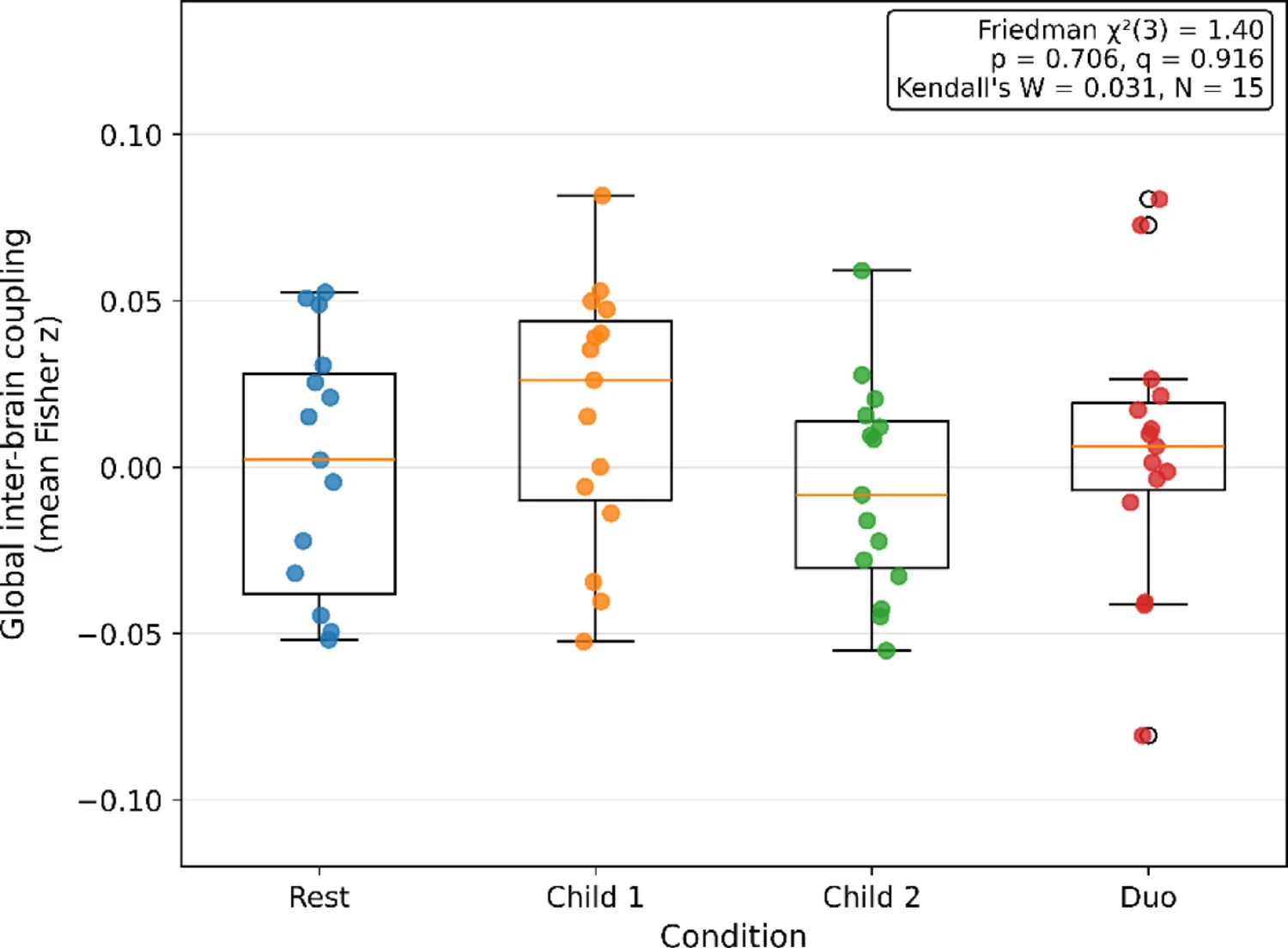
Global HbO inter-brain coupling across experimental conditions. Boxplots show dyad-level global Pearson coupling (mean Fisher z) during Rest, Child 1, Child 2, and Duo. Boxplots indicate medians, interquartile ranges, and variability across dyads

Despite the pronounced behavioral differentiation of Duo, global HbO IBC based on Fisher-transformed Pearson correlations did not differ across Rest, Child 1, Child 2, and Duo, Friedman chi-square(3) = 1.40, p = 0.706, FDR q = 0.916, Kendall's W = 0.031 (Fig. 3). The result was unchanged after excluding the first 8 s of each block, chi-square(3) = 1.40, p = 0.706, q = 0.806, W = 0.031. No ROI-specific Pearson summary showed a corrected condition effect for HbO or HbR, and no individual cross-brain channel pair survived FDR correction in the Duo-Rest, Duo-Child 1, or Duo-Child 2 contrasts.

Global IBC, as measured by large-scale cross-brain correlation, did not differ significantly between rest, single-agent problem solving, observation, and collaborative interaction. During Duo, global HbO coupling did not differ between real dyads (mean Fisher z = 0.0046) and temporally matched pseudodyads (mean = 0.0033), permutation p = 0.890, FDR q = 0.990 (Supplementary Table S5). Full omnibus and regional statistics are provided in Supplementary Table S4.

### Brain-behavior associations

**Fig. 4:**
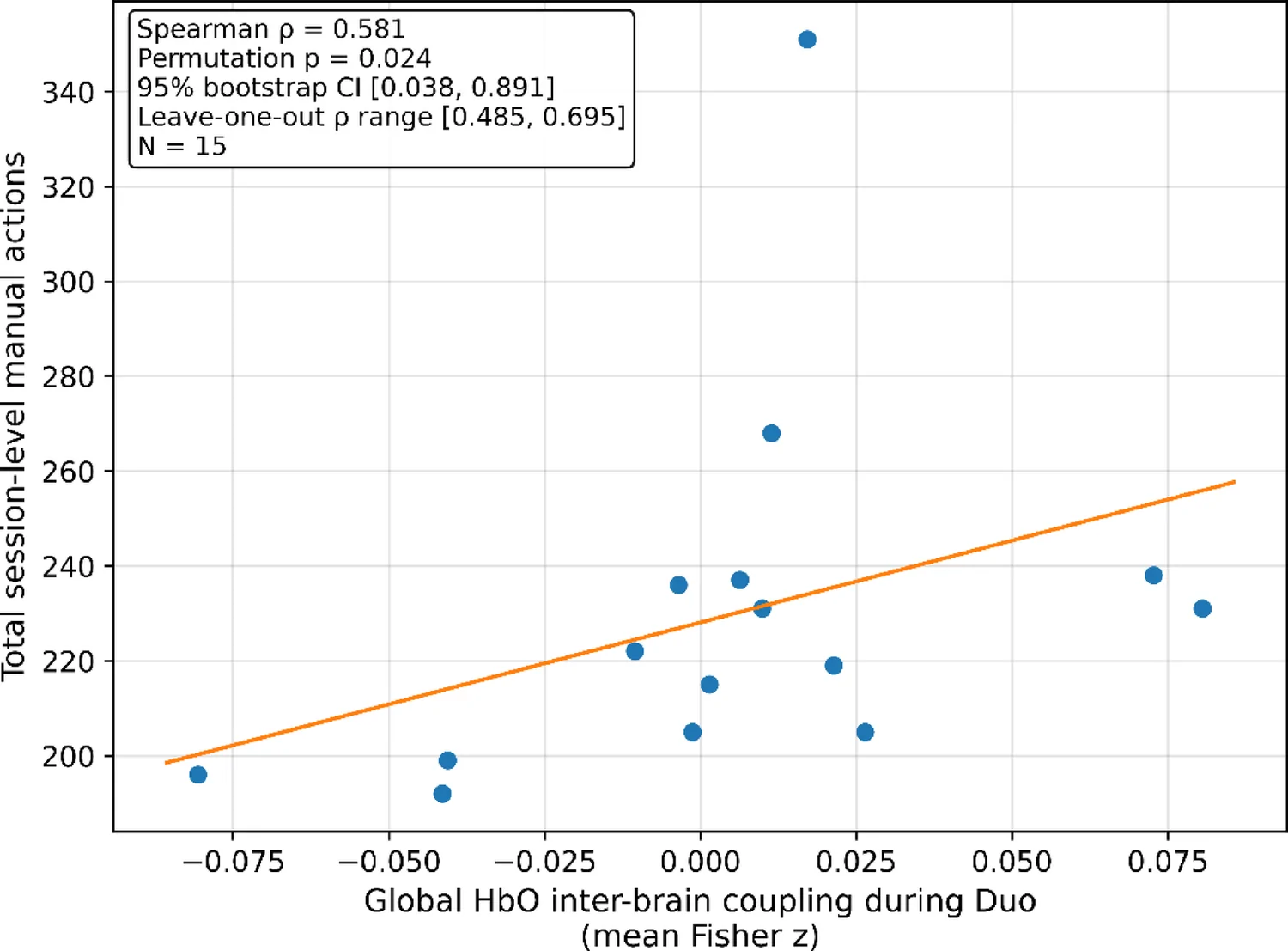
Exploratory association between inter-brain coupling and manual activity. Global HbO coupling during Duo was positively associated with total session-level manual actions (Spearman’s ρ = 0.581; permutation p = 0.024; 95% bootstrap CI [0.038, 0.891]; N = 15). The fitted line is included for visualization only

The association reported in the initial analysis between global HbO Pearson IBC during Duo and total session-level manual activity was reproduced (Spearman rho = 0.581, permutation p = 0.024, bootstrap 95% CI [0.038, 0.891], N = 15; Fig. 4). Four features support retaining this result as a hypothesis-generating observation: the coefficient had a relevant magnitude; the bootstrap interval excluded zero. It remained positive in every leave-one-dyad-out analysis (rho range = 0.485-0.695), and exclusion of the first 8 s produced a similar coefficient, rho = 0.538, permutation p = 0.041. The association did not survive FDR correction across the expanded reviewer-requested brain–behavior family (q = 0.131), and the trimmed bootstrap interval included zero (95% CI [− 0.007, 0.832]; q = 0.171; Supplementary Tables S6 and S7).

When behavior was restricted to the collaborative blocks, the association was smaller for Duo action frequency (rho = 0.360; permutation p = 0.184; q = 0.403) and absent for cumulative Duo action duration (rho = 0.057; permutation p = 0.836; q = 0.894). The exploratory effect does not establish that coupling scales with reciprocal coordination. Instead, it suggests that block-aligned global covariance during collaboration may track a broader dyad-level tendency toward task engagement. The pseudodyad test and brain-behavior correlation address different estimands: the former tests whether the mean coupling of real pairs exceeds random pairs, whereas the latter tests whether between-dyad variation within real pairs covaries with behavior. A null group-level pseudodyad contrast, therefore, does not mathematically preclude a stable individual-difference association.

## Discussion

The present study investigated the neural correlates of collaborative problem solving in children using fNIRS hyperscanning, integrating global cross-brain summaries across the recorded montage with behavioral measures of engagement during a hands-on Tangram task. To our knowledge, this is one of the first demonstrations of two-child hyperscanning during a physically embodied, collaborative visuospatial task. This naturalistic design provides insights into how developing brains coordinate neural activity during real collaboration. Importantly, the present findings do not indicate that neural synchrony directly indexes learning success. Instead, the focal exploratory analysis suggests that block-wise global coupling during collaboration may covary with broader between-dyad differences in task-related manual engagement.

The present study yielded three main findings. First, dual-child fNIRS hyperscanning was feasible during a physically interactive and behaviorally demanding task. Second, the Duo condition produced a marked increase in manual-action frequency and duration, confirming that collaboration constituted a behaviorally distinct experimental state. Third, this behavioral differentiation was not accompanied by a reliable condition-level increase in global or regional inter-brain coupling. A focal exploratory analysis nevertheless showed that between-dyad variation in global Pearson coupling during Duo covaried with broader session-level manual activity. This association was supported by permutation inference, a bootstrap interval excluding zero, and leave-one-dyad-out stability, but did not survive correction across the expanded brain-behavior family and was weaker for collaboration-specific activity.

### Dual-child hyperscanning in naturalistic tasks

A primary contribution of this work is extending the application of fNIRS hyperscanning deployed in pairs of school-aged children engaged in a cognitively demanding, physically interactive task. This extends feasibility demonstrations previously shown in adult dyads during collaborative or competitive tasks (Baker et al. 2016; Czeszumski et al. 2021) and aligns with emerging evidence that fNIRS is particularly well suited to naturalistic child-child paradigms (Bonnaire et al. 2024; Zhou et al. 2025; Nelson et al. 2025). By preserving ecological validity while maintaining data quality, our findings support ongoing shifts in developmental neuroscience toward studying children in social contexts rather than artificial laboratory constraints. This methodological advance may facilitate broader adoption of hyperscanning in research on collaborative learning, joint attention, and peer interaction. In addition, these findings provide a step toward integrating hyperscanning approaches into classroom research frameworks, where embodied collaboration is central to learning dynamics.

### Neural activation during collaborative problem solving

The revised activation analyses did not provide confirmatory evidence of posterior-parietal recruitment during collaboration. The prespecified posterior-parietal ROI did not differ significantly from baseline after dyad-level inference, and the positive effect observed at channel 17 did not survive channel-wise FDR correction. Nevertheless, its moderate effect size, anatomically plausible location, and opposite-direction HbO-HbR pattern identify a provisional spatial hypothesis for future studies.

The peak channel (CH 17, CP4-CP6) was located over an approximate posterior-parietal region implicated in visuospatial processing, object manipulation, and the integration of perception and action. Its moderate effect size and anatomical plausibility therefore identify a provisional spatial hypothesis for future studies. However, the effect was sensitive to the removal of individual dyads, and the available montage and sample do not permit precise localization or attribution to a specific functional network. Posterior parietal regions are widely implicated in integrating perception and action during object manipulation and spatially guided behavior (Wilson and Knoblich 2005; Sebanz et al. 2006; Glenberg 2008), and the parieto-frontal mirror system has been involved in executing actions, predicting, and interpreting others' physical actions in shared space (Rizzolatti and Sinigaglia 2010). The Tangram task required participants to monitor the shared workspace and provided opportunities to adjust their actions in relation to their partner. The observed activation is therefore consistent with increased demands on visuomotor activity during collaborative manipulation compared to baseline.

Within the joint action literature, coordinated behavior has been proposed to rely on the alignment between observed and executed movements (Wilson and Knoblich 2005; Sebanz et al. 2006). In this framework, posterior parietal activity may reflect the integration of visual information about a partner’s actions with one’s own motor planning processes, supporting temporally organized interaction. Importantly, the present data do not allow attribution to a specific functional network. fNIRS measures superficial hemodynamic signals with limited spatial specificity and substantial inter-channel covariance (Pinti et al. 2020; Yücel et al. 2021). Therefore, the current findings are compatible with accounts in which social coordination emerges from domain-general sensorimotor coupling during joint behavior (Hari et al. 2015; Redcay and Schilbach 2019).

### Condition-level stability in inter-brain connectivity

Contrary to initial expectations, global IBC did not vary significantly across experimental conditions. Rather than contradicting prior hyperscanning findings in adults (Nozawa et al. 2016), this result suggests that inter-brain coupling is not necessarily a categorical marker of task type. Instead, it may depend on the temporal structure of interaction, consistent with evidence that IBC reflects dynamic social alignment over time (Dai et al. 2018; Wass et al. 2020).

Several factors may contribute to the absence of condition-level effects. First, developmental variability may increase heterogeneity in neural coordination across dyads, making global condition contrasts less sensitive in children (Sheridan et al. 2014). Second, the large-scale, channel-agnostic IBC metric used here summarizes widespread cross-brain covariance, which likely includes both interaction-related and condition-invariant components. Such aggregation can reduce sensitivity to categorical contrasts while potentially retaining sensitivity to stable between-dyad differences.

Accordingly, the absence of a condition-level effect does not exclude meaningful between-dyad variation in coupling during collaboration. The focal brain–behavior association suggests that a block-wise global Pearson measure may capture differences among dyads that are not expressed as a uniform categorical contrast between experimental conditions. However, the current data do not establish that IBC is a general index of manual activity, because the association was exploratory, did not survive the expanded multiplicity correction, and was weaker when behavior was restricted to the Duo condition. The condition-level and correlational analyses therefore address complementary questions: the former asks whether collaboration produces a uniform increase across dyads, whereas the latter asks whether variation among real dyads covaries with behavioral engagement.

### Exploratory association between block-wise inter-brain coupling and dyad-level manual engagement

During the collaborative condition, the IBC was positively associated with behavioral engagement, specifically the number of actions performed on the Tangram pieces. This pattern is compatible with the possibility that between-dyad differences in behavioral engagement covary with block-wise neural coupling (Hasson et al. 2012; Redcay and Schilbach 2019). In developmental contexts, neural and behavioral synchrony have been discussed in relation to scaffolding and co-regulation (Hoehl et al. 2021). The present results indicate that dyads with greater overall manual activity tended to show higher global coupling during the Duo condition.

Importantly, this association suggests that dyads with greater overall task-related manual activity exhibited higher global block-wise cross-brain covariance during collaboration. Such a pattern is compatible with frameworks emphasizing embodied coordination and perception–action coupling (Glenberg 2008; Gallese 2014; Kontra et al. 2015) as well as interactive approaches to social neuroscience (Schilbach et al. 2013), without implying that identical cognitive processes occurred simultaneously in both participants.

Our results also relate to the distinction between co-action and joint action described in adult hyperscanning research. Previous studies indicate that simultaneous presence or parallel activity alone may be insufficient to produce robust inter-brain synchrony (Cui et al. 2012; Cheng et al. 2015). Because mean global coupling did not differ among observation, individual action, collaboration, and rest, the present data do not isolate social presence or collaboration as categorical determinants of the global IBC measure. The exploratory association was observed for the global Duo coupling score, but the behavioral variable showing the strongest relationship represented total session-level activity rather than a direct measure of continuous reciprocal adjustment. This observation is compatible with the proposal that social interaction can be understood in terms of coordinated action dynamics (Konvalinka and Roepstorff 2012).

From a developmental perspective, the exploratory association raises the possibility that stable differences in dyad-level engagement are related to inter-brain measures during peer collaboration. Given that collaboration supports learning in children (Warneken 2018; Tomasello 2019), these findings may help constrain hypotheses about when alignment-like neural signatures emerge during peer interaction, potentially during hands-on, jointly coordinated activity. Accordingly, the results motivate future classroom research but should not be interpreted as direct evidence for learning.

### Limitations and future directions

Several methodological considerations should be noted when interpreting the present findings. First, although fNIRS is relatively tolerant to motion, the naturalistic, hands-on nature of the task inevitably elicited head movements. While careful signal processing was applied, managing motion artifacts remains an inherent challenge in unconstrained experimental settings. Second, our participants' age range spans a period of significant neurocognitive development, which likely introduced natural variability in executive function, social cognition, and collaborative skills. Third, the sensitivity analysis indicated that the sample could reliably detect only large effects. It limited our capacity to model more subtle variables, such as developmental trajectories or sex-composition effects. Additionally, dyads were formed by classmates; while this reflects typical peer interactions, relationship closeness was not formally quantified. Pre-existing familiarity may have facilitated collaboration, thereby increasing between-dyad heterogeneity. Moreover, the inherent variation in Tangram puzzle difficulty may have resulted in fluctuating cognitive demands across dyads and conditions. Regarding behavioral analysis, manual activity was coded by a single investigator, precluding the assessment of inter-rater reliability. Finally, our design deliberately prohibited speech and intentional gestures to reduce communication-related confounds. While this was necessary to isolate the effects of shared manual action, it constrained the ecological validity of the task and may have attenuated neural coupling that would otherwise emerge during unconstrained peer communication. The present conclusions therefore apply specifically to nonverbal, object-mediated collaboration.

Given the partial sensitivity of the CH17 finding to the multiple-comparisons strategy, we treat this localization as a promising but provisional result. The moderate effect size and anatomical plausibility motivate targeted follow-up studies to confirm localized posterior-parietal recruitment during collaborative embodied tasks. Furthermore, the lack of independent measures of executive function or social-cognitive skills limited our ability to connect neural coupling to individual developmental differences. Lastly, the small sample size limits statistical power and the generalizability of the findings.

Moreover, a recent meta-analysis suggests that children's age and brain regions are significant predictors of effect size in parent–child research (Zhao et al. 2024). Future research should involve larger, developmentally focused samples and incorporate additional behavioral assessments to refine and expand these findings. It should also examine developmental trajectories by including a broader age range of children and comparing contexts, such as collaborative versus competitive settings, to evaluate how neural response coupling varies across tasks.

## Conclusion

The present study demonstrates that nonverbal, hands-on collaboration between child peers can be behaviorally pronounced without producing a correspondingly robust condition-level increase in global inter-brain coupling. Block-aligned Fisher-transformed Pearson analyses, regional summaries, channel-pair correction, carry-over controls, and pseudodyads did not reliably distinguish collaboration from rest, individual action, or observation. Nevertheless, the exploratory association between Duo coupling and broader manual activity showed a relevant magnitude, a bootstrap interval that excluded zero, stability in leave-one-dyad-out analyses, and a similar effect after removal of early block periods. Because it did not survive multiplicity correction and was weaker for collaboration-specific behavior, the association should be interpreted as hypothesis-generating rather than confirmatory. Accordingly, the present results contribute to ongoing efforts to characterize the relationship between interpersonal neural measures and real-world child-child interaction. These findings inform future research and identify testable boundary conditions for how block-aligned cross-brain covariance relates to dyad-level engagement.

## Supplementary Information

Below is the link to the electronic supplementary material.

Supplementary Material 1

## Data Availability

Due to ethical constraints, the datasets generated during the current study are available from the corresponding author upon reasonable request.
