# Supplementary material for: Inter-brain coupling during naturalistic collaborative Tangram solving in child dyads: an exploratory fNIRS hyperscanning study
Source: Brain Struct Funct. 2026 Aug 29;231(7):128. doi: 10.1007/s00429-026-03187-5 (PMC13526130; doi:10.1007/s00429-026-03187-5)
Supplement: Supplementary file 1 [file 429_2026_3187_MOESM1_ESM.docx]

**Supplementary Material**

**Inter-brain coupling during naturalistic collaborative Tangram solving**

**in child dyads: an exploratory fNIRS hyperscanning study**

Raimundo da Silva Soares Junior and João Ricardo Sato

S1. Supplementary Data

Thirty participant signal files and 30 corresponding timing files were available. Each signal file contained 9,766 rows and 144 numeric columns without a header. Timing files extended from 0 to 959.93856 s in 0.098304-s increments, corresponding to 10.172526 Hz. HbO occupied zero-based columns 96-119, and HbR occupied columns 120-143. Sixteen long-separation channels per participant were retained for connectivity analysis. Odd/even files corresponded to Child 1/Child 2 and formed 15 dyads.

S2. Behavioral event alignment and carry-over

The behavioral video began at the first active block, 30 s after the initial fixation block. A confirmed +30-s offset was therefore applied to the behavioral timestamps. Action episodes were assigned to conditions using their midpoint; duration was divided when an episode crossed a 30-s boundary. Dyad-level behavioral values are listed in Supplementary Table S1. Only 3.5% of mapped action episodes were assigned to Rest, and 95% of those episodes occurred within the first 7.8 s of a Rest block. This temporal concentration motivated a sensitivity analysis excluding the first 8 s of every block. The global condition effect and exploratory brain-behavior coefficient were similar after this exclusion (Supplementary Table S6; Supplementary Figure S1).

**Supplementary Table S1.** Dyad-level behavioral measures.

| **Dyad** | **Solved** | **Total actions** | **Asymmetry** | **Child 1 actions** | **Child 2 actions** | **Duo actions** | **Child 1 duration** | **Child 2 duration** | **Duo duration** |
| --- | --- | --- | --- | --- | --- | --- | --- | --- | --- |
| A | 4 | 205 | 0.044 | 60 | 57 | 82 | 113.2 | 115.8 | 152.0 |
| B | 3 | 231 | 0.134 | 57 | 54 | 91 | 119.6 | 122.9 | 132.6 |
| C | 7 | 268 | 0.261 | 74 | 70 | 110 | 128.4 | 104.0 | 136.5 |
| D | 3 | 236 | 0.017 | 60 | 66 | 103 | 145.1 | 140.1 | 172.3 |
| E | 3 | 231 | 0.065 | 57 | 59 | 110 | 150.7 | 129.8 | 212.4 |
| F | 3 | 215 | 0.163 | 45 | 72 | 93 | 96.1 | 124.5 | 125.4 |
| G | 2 | 237 | 0.291 | 77 | 58 | 91 | 147.5 | 143.9 | 151.8 |
| H | 2 | 222 | 0.018 | 62 | 63 | 97 | 155.7 | 146.8 | 187.3 |
| I | 3 | 192 | 0.198 | 55 | 51 | 83 | 140.8 | 133.5 | 177.6 |
| J | 3 | 196 | 0.133 | 56 | 59 | 72 | 114.3 | 118.0 | 130.7 |
| K | 4 | 238 | 0.025 | 59 | 70 | 103 | 140.4 | 156.2 | 201.6 |
| L | 4 | 199 | 0.075 | 55 | 63 | 65 | 166.5 | 149.2 | 139.5 |
| M | 4 | 205 | 0.268 | 46 | 66 | 81 | 94.7 | 137.7 | 139.5 |
| N | 10 | 351 | 0.077 | 91 | 97 | 157 | 136.7 | 138.9 | 175.1 |
| O | 7 | 219 | 0.142 | 63 | 62 | 81 | 105.1 | 144.8 | 139.5 |

S3. Activation analysis details

Confirmatory activation analyses used dyad-averaged beta values. Holm correction was applied across the three prespecified ROIs within each contrast. Channel-wise analyses were corrected across 24 channels using the Benjamini-Hochberg false discovery rate. Complete HbO and HbR ROI estimates are reported in Supplementary Table S2. The exploratory channel-17 result, participant-level sensitivity analysis, HbR direction, and confidence intervals are summarized in Supplementary Table S3.

**Supplementary Table S2.** Dyad-level ROI activation results.

| **Chrom.** | **Contrast** | **ROI** | **Mean beta** | **SD** | **t(14)** | **p** | **Holm p** | **dz** |
| --- | --- | --- | --- | --- | --- | --- | --- | --- |
| HbO | Active > baseline | dlPFC | 24.28 | 71.20 | 1.32 | 0.208 | 0.623 | 0.34 |
| HbO | Active > baseline | Posterior parietal | 32.51 | 101.37 | 1.24 | 0.235 | 0.623 | 0.32 |
| HbO | Active > baseline | rTPJ | -2.33 | 44.31 | -0.20 | 0.841 | 0.841 | -0.05 |
| HbO | Observation > baseline | dlPFC | 18.54 | 37.05 | 1.94 | 0.073 | 0.219 | 0.50 |
| HbO | Observation > baseline | Posterior parietal | 11.11 | 60.44 | 0.71 | 0.488 | 0.976 | 0.18 |
| HbO | Observation > baseline | rTPJ | 0.91 | 33.80 | 0.10 | 0.919 | 0.976 | 0.03 |
| HbO | Duo > baseline | dlPFC | 15.75 | 47.30 | 1.29 | 0.218 | 0.654 | 0.33 |
| HbO | Duo > baseline | Posterior parietal | 18.24 | 61.48 | 1.15 | 0.270 | 0.654 | 0.30 |
| HbO | Duo > baseline | rTPJ | -1.79 | 25.23 | -0.28 | 0.787 | 0.787 | -0.07 |
| HbO | Duo > Active | dlPFC | -8.52 | 24.22 | -1.36 | 0.194 | 0.583 | -0.35 |
| HbO | Duo > Active | Posterior parietal | -14.26 | 40.79 | -1.35 | 0.197 | 0.583 | -0.35 |
| HbO | Duo > Active | rTPJ | 0.54 | 22.91 | 0.09 | 0.928 | 0.928 | 0.02 |
| HbO | Duo > Observation | dlPFC | -2.79 | 23.28 | -0.46 | 0.650 | 1.000 | -0.12 |
| HbO | Duo > Observation | Posterior parietal | 7.13 | 16.93 | 1.63 | 0.125 | 0.375 | 0.42 |
| HbO | Duo > Observation | rTPJ | -2.70 | 22.12 | -0.47 | 0.644 | 1.000 | -0.12 |
| HbO | Active > Observation | dlPFC | 5.74 | 43.35 | 0.51 | 0.616 | 1.000 | 0.13 |
| HbO | Active > Observation | Posterior parietal | 21.40 | 48.56 | 1.71 | 0.110 | 0.330 | 0.44 |
| HbO | Active > Observation | rTPJ | -3.24 | 32.94 | -0.38 | 0.709 | 1.000 | -0.10 |
| HbR | Active > baseline | dlPFC | -9.95 | 32.32 | -1.19 | 0.253 | 0.759 | -0.31 |
| HbR | Active > baseline | Posterior parietal | -2.99 | 31.74 | -0.37 | 0.720 | 0.759 | -0.09 |
| HbR | Active > baseline | rTPJ | 4.27 | 17.97 | 0.92 | 0.373 | 0.759 | 0.24 |
| HbR | Observation > baseline | dlPFC | -4.15 | 18.46 | -0.87 | 0.398 | 0.821 | -0.22 |
| HbR | Observation > baseline | Posterior parietal | -4.78 | 33.89 | -0.55 | 0.594 | 0.821 | -0.14 |
| HbR | Observation > baseline | rTPJ | 6.08 | 20.68 | 1.14 | 0.274 | 0.821 | 0.29 |
| HbR | Duo > baseline | dlPFC | -5.51 | 20.41 | -1.05 | 0.314 | 0.941 | -0.27 |
| HbR | Duo > baseline | Posterior parietal | -1.15 | 16.32 | -0.27 | 0.789 | 0.941 | -0.07 |
| HbR | Duo > baseline | rTPJ | 2.90 | 10.92 | 1.03 | 0.321 | 0.941 | 0.27 |
| HbR | Duo > Active | dlPFC | 4.44 | 12.02 | 1.43 | 0.174 | 0.523 | 0.37 |
| HbR | Duo > Active | Posterior parietal | 1.84 | 18.06 | 0.39 | 0.699 | 1.000 | 0.10 |
| HbR | Duo > Active | rTPJ | -1.37 | 10.22 | -0.52 | 0.612 | 1.000 | -0.13 |
| HbR | Duo > Observation | dlPFC | -1.36 | 3.71 | -1.42 | 0.178 | 0.534 | -0.37 |
| HbR | Duo > Observation | Posterior parietal | 3.63 | 23.63 | 0.59 | 0.562 | 0.832 | 0.15 |
| HbR | Duo > Observation | rTPJ | -3.18 | 14.70 | -0.84 | 0.416 | 0.832 | -0.22 |
| HbR | Active > Observation | dlPFC | -5.80 | 14.43 | -1.56 | 0.142 | 0.425 | -0.40 |
| HbR | Active > Observation | Posterior parietal | 1.79 | 20.43 | 0.34 | 0.740 | 1.000 | 0.09 |
| HbR | Active > Observation | rTPJ | -1.81 | 16.80 | -0.42 | 0.683 | 1.000 | -0.11 |

**Supplementary Table S3.** Channel 17 exploratory activation and sensitivity.

| **Chrom.** | **Unit** | **Mean beta** | **t** | **p** | **FDR q** | **dz** | **95% CI** |
| --- | --- | --- | --- | --- | --- | --- | --- |
| HbO | 15 dyads | 21.14 | 2.16 | 0.049 | 0.594 | 0.56 | [0.11, 42.18] |
| HbO | 30 participants | 21.14 | 2.78 | 0.009 | 0.227 | 0.51 | [5.58, 36.70] |
| HbR | 15 dyads | -9.44 | -1.70 | 0.110 | 0.557 | -0.44 | [-21.31, 2.44] |

S4. Block-aligned Pearson inter-brain connectivity

Pearson coefficients were calculated separately for each 30-s block, transformed using Fisher's r-to-z transformation, and averaged across the eight repetitions of each condition. Negative coefficients were retained. The global metric was the mean of all 256 cross-brain coefficients; additional metrics summarized homologous channels and within- and between-ROI connections. Pearson correlation was selected because it estimates zero-lag covariance entirely within the command-defined 30-s windows. Wavelet transform coherence was not used as the principal estimator because commonly examined slow hemodynamic periods can equal or exceed the block duration. The temporal support of slower wavelet scales consequently extends across adjacent conditions and limits condition specificity (Grinsted et al., 2004; Torrence & Compo, 1998). Omnibus tests for all global and regional Pearson summaries are reported in Supplementary Table S4.

**Supplementary Table S4.** Pearson IBC omnibus condition tests. PPC = posterior parietal cortex; bidirectional ROI pairs combine both cross-region directions.

| **Chrom.** | **Trim, s** | **Metric** | **chi-square(3)** | **p** | **FDR q** | **Kendall W** |
| --- | --- | --- | --- | --- | --- | --- |
| HbO | 0 | Global | 1.40 | 0.706 | 0.916 | 0.031 |
| HbO | 0 | Homologous | 0.44 | 0.932 | 0.932 | 0.010 |
| HbO | 0 | PPC-PPC | 5.32 | 0.150 | 0.458 | 0.118 |
| HbO | 0 | PPC-rTPJ | 1.96 | 0.581 | 0.916 | 0.044 |
| HbO | 0 | dlPFC-PPC | 9.08 | 0.028 | 0.226 | 0.202 |
| HbO | 0 | dlPFC-dlPFC | 1.00 | 0.801 | 0.916 | 0.022 |
| HbO | 0 | dlPFC-rTPJ | 2.44 | 0.486 | 0.916 | 0.054 |
| HbO | 0 | rTPJ-rTPJ | 5.00 | 0.172 | 0.458 | 0.111 |
| HbO | 8 | Global | 1.40 | 0.706 | 0.806 | 0.031 |
| HbO | 8 | Homologous | 1.64 | 0.650 | 0.806 | 0.036 |
| HbO | 8 | PPC-PPC | 8.20 | 0.042 | 0.336 | 0.182 |
| HbO | 8 | PPC-rTPJ | 1.64 | 0.650 | 0.806 | 0.036 |
| HbO | 8 | dlPFC-PPC | 1.80 | 0.615 | 0.806 | 0.040 |
| HbO | 8 | dlPFC-dlPFC | 2.68 | 0.444 | 0.806 | 0.060 |
| HbO | 8 | dlPFC-rTPJ | 0.04 | 0.998 | 0.998 | 0.001 |
| HbO | 8 | rTPJ-rTPJ | 4.84 | 0.184 | 0.736 | 0.108 |
| HbR | 0 | Global | 6.44 | 0.092 | 0.245 | 0.143 |
| HbR | 0 | Homologous | 2.44 | 0.486 | 0.650 | 0.054 |
| HbR | 0 | PPC-PPC | 8.84 | 0.031 | 0.245 | 0.196 |
| HbR | 0 | PPC-rTPJ | 2.76 | 0.430 | 0.650 | 0.061 |
| HbR | 0 | dlPFC-PPC | 7.16 | 0.067 | 0.245 | 0.159 |
| HbR | 0 | dlPFC-dlPFC | 1.96 | 0.581 | 0.650 | 0.044 |
| HbR | 0 | dlPFC-rTPJ | 1.64 | 0.650 | 0.650 | 0.036 |
| HbR | 0 | rTPJ-rTPJ | 2.36 | 0.501 | 0.650 | 0.052 |
| HbR | 8 | Global | 5.24 | 0.155 | 0.440 | 0.116 |
| HbR | 8 | Homologous | 4.68 | 0.197 | 0.440 | 0.104 |
| HbR | 8 | PPC-PPC | 3.88 | 0.275 | 0.440 | 0.086 |
| HbR | 8 | PPC-rTPJ | 1.80 | 0.615 | 0.615 | 0.040 |
| HbR | 8 | dlPFC-PPC | 1.96 | 0.581 | 0.615 | 0.044 |
| HbR | 8 | dlPFC-dlPFC | 1.96 | 0.581 | 0.615 | 0.044 |
| HbR | 8 | dlPFC-rTPJ | 4.92 | 0.178 | 0.440 | 0.109 |
| HbR | 8 | rTPJ-rTPJ | 3.96 | 0.266 | 0.440 | 0.088 |

S5. Temporally matched pseudodyad controls

Pseudodyads were generated using 5,000 random derangements that paired Child 1 from each real dyad with Child 2 from a different dyad. This procedure preserved the full temporal sequence, audio-trigger structure, and condition labels while disrupting the real partnership. Global Pearson comparisons are shown in Supplementary Table S5. Real dyads did not exceed the pseudodyad distribution during Duo, indicating that the group-average global score was not specific to the real partnership.

**Supplementary Table S5.** Global HbO Pearson real-versus-pseudodyad comparisons.

| **Metric** | **Condition** | **Real mean** | **Pseudo mean** | **Permutation p** | **FDR q** |
| --- | --- | --- | --- | --- | --- |
| Pearson | Rest | -0.0071 | -0.0021 | 0.582 | 0.778 |
| Pearson | Child1 | 0.0161 | 0.0055 | 0.311 | 0.836 |
| Pearson | Child2 | -0.0065 | -0.0027 | 0.685 | 0.865 |
| Pearson | Duo | 0.0046 | 0.0033 | 0.890 | 0.990 |

S6. Carry-over sensitivity

All Pearson analyses were repeated after excluding the first 8 s of every 30-s block. As summarized in Supplementary Table S6 and Supplementary Figure S1, trimming did not reveal a condition effect and yielded a brain-behavior coefficient similar in magnitude to the full-block estimate. The similarity supports the view that the exploratory association was not driven solely by immediate transition periods, although the trimmed bootstrap interval included zero and the result remained uncorrected for multiplicity.

**Supplementary Table S6.** Carry-over sensitivity of the global Pearson results.

| **Analysis** | **Full 30-s blocks** | **First 8 s excluded** |
| --- | --- | --- |
| Global HbO Pearson condition effect | chi-square(3) = 1.40; p = .706; FDR q = .916; W = .031 | chi-square(3) = 1.40; p = .706; FDR q = .806; W = .031 |
| Duo global HbO IBC x total actions | rho = .581; permutation p = .024; FDR q = .131; 95% CI [.038, .891] | rho = .538; permutation p = .041; FDR q = .171; 95% CI [-.007, .832] |

Supplementary Figure S1. Mean global HbO Pearson IBC using full blocks and after exclusion of the first 8 s. The condition profile was similar under both definitions.

S7. Brain-behavior associations

Selected HbO Pearson brain-behavior results are reported in Supplementary Table S7. The full-block association between global Duo IBC and total manual activity had a relevant magnitude (rho = .581), a bootstrap interval excluding zero, and remained positive in every leave-one-dyad-out analysis (rho range = .485-.695). The 8-s-trimmed coefficient was similar (rho = .538). However, neither estimate survived FDR correction, and the association was weaker when activity was restricted to Duo action frequency and absent for Duo action duration. The finding is therefore retained as a hypothesis-generating relation between block-aligned cross-brain covariance and broader dyad-level engagement, not as evidence that coupling scales with interpersonal coordination.

**Supplementary Table S7.** Selected HbO Pearson brain-behavior associations during Duo.

| **Method** | **Trim** | **Metric** | **Behavior** | **rho** | **Perm. p** | **FDR q** | **Bootstrap 95% CI** |
| --- | --- | --- | --- | --- | --- | --- | --- |
| Pearson | 0 | Global | total_actions | 0.581 | 0.024 | 0.131 | [0.038, 0.891] |
| Pearson | 0 | Global | Duo_action_episodes | 0.360 | 0.184 | 0.403 | [-0.189, 0.756] |
| Pearson | 0 | Global | Duo_action_duration_s | 0.057 | 0.836 | 0.894 | [-0.512, 0.575] |
| Pearson | 8 | Global | total_actions | 0.538 | 0.041 | 0.171 | [-0.007, 0.832] |

Two unpredicted HbR Pearson associations in the Child 2 condition survived FDR within that condition (Supplementary Table S8). These analyses were not hypothesized, involved session-level behavioral variables, and were not accompanied by corrected condition-level coupling effects. They should therefore be treated as exploratory secondary observations rather than independent evidence for the primary hypotheses.

**Supplementary Table S8. Unpredicted exploratory HbR associations in Child 2.**

| **Metric** | **Behavior** | **rho** | **Perm. p** | **FDR q** | **Bootstrap 95% CI** |
| --- | --- | --- | --- | --- | --- |
| dlPFC-PPC | total_actions | 0.732 | 0.0019 | 0.039 | [0.312, 0.925] |
| dlPFC-rTPJ | action_asymmetry | 0.746 | 0.0028 | 0.039 | [0.350, 0.933] |

S8. Statistical sensitivity

With 15 dyads, the study had 80% power only for large effects. The minimum detectable standardized paired/one-sample effect was dz = 0.778, and the minimum detectable absolute bivariate correlation was approximately .669 (Supplementary Table S9). Exact p values, effect sizes, bootstrap confidence intervals, leave-one-dyad-out ranges, and permutation-based inference were therefore emphasized throughout the revised manuscript.

**Supplementary Table S9.** Minimum detectable effects for N = 15 dyads.

| **Effect family** | **Two-sided alpha** | **Target power** | **Minimum detectable effect** |
| --- | --- | --- | --- |
| Paired/one-sample standardized mean | .05 | .80 | dz = 0.778 |
| Bivariate correlation | .05 | .80 | \|rho\| approximately 0.669 |

**Supplementary References**

Burgess, A. P. (2013). On the interpretation of synchronization in EEG hyperscanning studies: A cautionary note. Frontiers in Human Neuroscience, 7, 881.

Grinsted, A., Moore, J. C., & Jevrejeva, S. (2004). Application of the cross wavelet transform and wavelet coherence to geophysical time series. Nonlinear Processes in Geophysics, 11, 561-566.

Huppert, T. J. (2016). Commentary on the statistical properties of noise and its implications for general linear models in functional near-infrared spectroscopy. Neurophotonics, 3(1), 010401.

Torrence, C., & Compo, G. P. (1998). A practical guide to wavelet analysis. Bulletin of the American Meteorological Society, 79(1), 61-78.

Warneken, F. (2018). How children solve the two challenges of cooperation. Annual Review of Psychology, 69, 205-229.
